# Hepatic Stellate Cells Enhance Liver Cancer Progression by Inducing Myeloid-Derived Suppressor Cells through Interleukin-6 Signaling

**DOI:** 10.3390/ijms20205079

**Published:** 2019-10-13

**Authors:** Ching-Chuan Hsieh, Chien-Hui Hung, Meihua Chiang, Yu-Chin Tsai, Jie-Teng He

**Affiliations:** 1 Graduate Institute of Clinical Medical Sciences, College of Medicine, Chang-Gung University, Taoyuan 333, Taiwan; hungc01@mail.cgu.edu.tw; 2 Division of General Surgery, Chang-Gung Memorial Hospital, Chiayi 613, Taiwan; maha0210@gmail.com (M.C.); anael1228@gmail.com (Y.-C.T.); r90225029@ntu.edu.tw (J.-T.H.); 3 Division of Infectious Diseases, Chang-Gung Memorial Hospital, Chiayi 613, Taiwan

**Keywords:** hepatic stellate cells, myeloid-derived suppressor cells, liver cancer, interleukin-6

## Abstract

The tumor microenvironment, which consists of fibroblasts, smooth muscle cells, endothelial cells, immune cells, epithelial cells, and extracellular matrices, plays a crucial role in tumor progression. Hepatic stellate cells (HSCs), a class of unique liver stromal cells, participate in immunomodulatory activities by inducing the apoptosis of effector T-cells, generation of regulatory T-cells, and development of myeloid-derived suppressor cells (MDSCs) to achieve long-term survival of islet allografts. This study provides in vitro and in vivo evidences that HSCs induce the generation of MDSCs to promote hepatocellular carcinoma (HCC) progression through interleukin (IL)-6 secretion. HSC-induced MDSCs highly expressed inducible nitric oxide synthase (iNOS) and arginase 1 mRNA and presented potent inhibitory T-cell immune responses in the tumor environment. Wild-type HSC-induced MDSCs expressed lower levels of CD40, CD86, and MHC II, and a higher level of B7-H1 surface molecules, as well as increased the production of iNOS and arginase I compared with MDSCs induced by IL-6-deficient HSCs in vitro. A murine-transplanted model of the liver tumor showed that HCCs cotransplanted with HSCs could significantly enhance the tumor area and detect more MDSCs compared with HCCs alone or HCCs cotransplanted with HSCs lacking IL-6. In conclusion, the results indicated that MDSCs are induced mainly by HSCs through IL-6 signaling and produce inhibitory enzymes to reduce T-cell immunity and then promote HCC progression within the tumor microenvironment. Therapies targeting the pathway involved in MDSC production or its immune-modulating pathways can serve as an alternative immunotherapy for HCC.

## 1. Introduction

The tumor microenvironment, which consists of several host-derived cell types, including fibroblasts, smooth muscle cells, endothelial cells, immune cells, and epithelial cells, and the extracellular matrix, plays a crucial role in tumor progression [1]. Tumors have developed various mechanisms to evade a host’s immune response and have generated a suppressive network. For example, cancer-activated fibroblasts play a key role in cancer progression by secreting soluble factors, such as growth factors and inflammatory mediators, and by producing extracellular matrix proteins and their proteases [2].

Hepatic stellate cells (HSCs), a class of unique stromal cells, participate in reparative processes after liver injury [3,4,5]. Activated HSCs produce growth factor, extracellular matrices, and cytokines [4]. In addition, HSCs possess potent immunomodulatory activity when activated. In previous studies, HSCs markedly suppressed allogeneic T-cell immune responses through the induction of T-cell apoptosis, which was partially mediated by B7-H1 expressed on HSCs [6], and preferentially expanded allogeneic CD4^+^, CD25^+^, and FoxP3^+^ regulatory T-cells [7]. In addition, cotransplanted HSCs with allogeneic islets effectively protected islet allografts from rejection by secreting complement component 3 to induce the generation of myeloid-derived suppressive cells (MDSCs) [8,9].

MDSCs are an intrinsic part of the myeloid cell lineage and are a heterogeneous population consisting of myeloid cell progenitors and myeloid cell precursors. In healthy individuals, immature myeloid cells (IMCs) generated in the bone marrow (BM) rapidly differentiate into mature granulocytes, macrophages, or dendritic cells (DCs). In pathological conditions, such as cancer, infection, trauma, and some autoimmune disorders, a partial block of the development of IMCs into mature myeloid cells results in the expansion of this population [10].

Similar to many other types of cancer, hepatocellular carcinoma (HCC) have devised mechanisms to evade a host’s immune responses, such as an increase in regulatory T-cells and defects in antigen-presenting cells [11]. A study performed ex vivo isolated a new subset of MDSCs that was significantly increased in the peripheral blood and tumor of patients with HCC [12]. Interleukin (IL)-6 is a major proinflammatory cytokine and is recognized as a key regulator of immunosuppression in advanced cancers [13,14,15]. Evidence supports the concept that chronic inflammation promotes the development and progression of cancers, and immune suppression can be one of the mechanisms underlying the fact that inflammation facilitates tumor progression [15,16].

In this study, we discerned both in vivo and in vitro evidence that HSCs preferentially induce the generation of MDSCs under the HCC environment, which in turn promotes HCC progression. These effects are dependent on the IL-6 signaling pathway in HSCs and are mediated by a soluble factor.

## 2. Results

### 2.1. Features of HSC-Conditioned Monocytes Resemble MDSCs In Vitro

**Figure 1:**
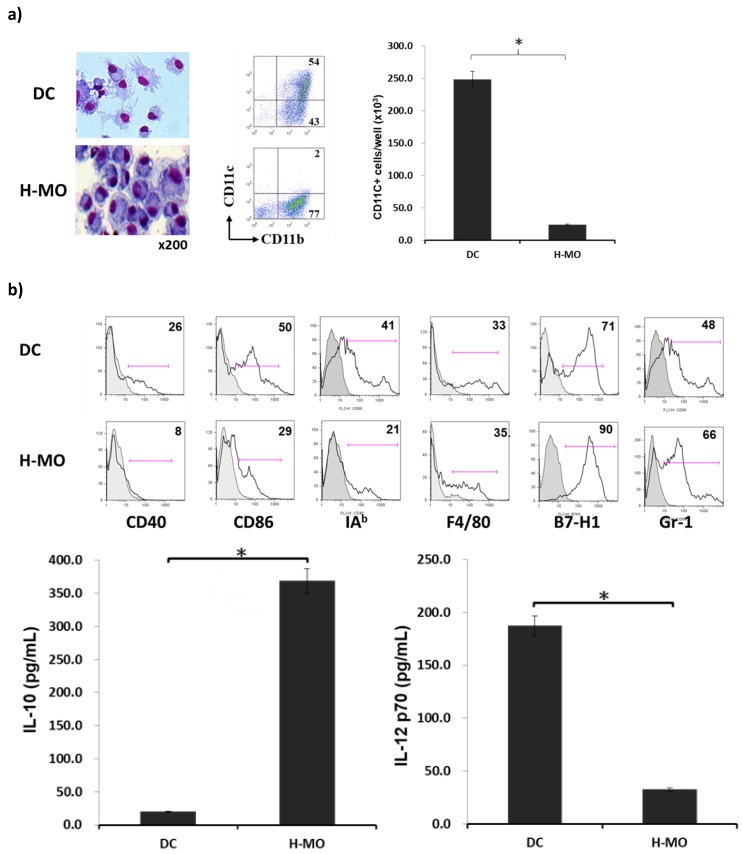

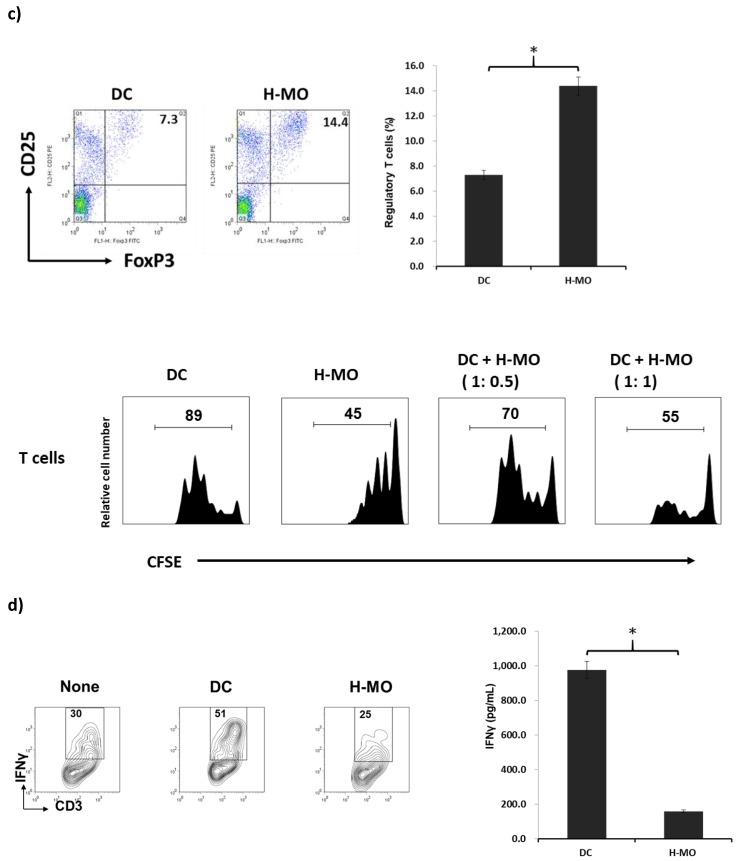
Effect of HSCs on BM-derived monocyte propagation in vitro. HSCs (B6) were added (HSCs/BM cells = 1:40) at the beginning of the culture of B6 BM cells (2 × 10^6^ cells/well) in the presence of mouse recombinant GM-CSF (8 ng/mL) for 5 days. The culture in the absence of HSCs served as the control (DC). (**a**) Cells were stained with Giemsa for morphology examination. Cells were stained for surface molecules CD11b and CD11c and analyzed through flow cytometry. The absolute numbers of CD11c^+^ cells/well were calculated (*n* = 3) and expressed as the mean ± 1 SD (* *p* < 0.05). (**b**) Cells were stained for CD40, CD86, IAb (MHC II), F4/80, B7-H1, and Gr-1, and analyzed through flow cytometry. The flow histograms represent the expression of the indicated surface molecules. The levels of IL-10 and IL-12 p70 were measured in the culture supernatant by using ELISA (* *p* < 0.05). (**c**) Expression of regulatory T-cells (CD4^+^/CD25^+^/Foxp3^+^) was assayed through intracellular staining with specific mAbs and analyzed through flow cytometry. Numbers represent the percentage of double-positive cells in the CD4^+^ T-cell subset. The bar graph shows the ratio of Treg cells differentiated from the DC or H-MO group (upper panel; * *p* < 0.05). CFSE-labeled BALB/c spleen T-cells were cultured with B6 DCs or H-MOs at a ratio of 20:1 for 3 days. B6 H-MOs were added at the beginning into the culture at a DC/H-MO ratio of 1:0.5 or 1:1. The proliferation of T-cells was determined through CFSE dilution gated in the CD3 population (lower panel). (**d**) Expression of IFN-γ from stimulated allogeneic T-cells was determined through intracellular staining with specific mAbs or the cultured supernatant by using ELISA (* *p* < 0.05). The data are representative of three separate experiments.

To determine the effects of HSCs on the generation of immune cells in vitro, HSCs were added at the beginning into the culture of BM cells in the presence of GM-CSF. Compared with DCs, HSC-conditioned monocytes (H-MOs) were differentiated into a small, round, and less cytoplasmic projection with an eccentric nucleus. H-MOs contained only 2% CD11c^+^ cells (CD11c^+^ cell number = 24 ± 14.9 × 10^3^/well), whereas DCs contained 54% CD11c^+^ cells (CD11c^+^ cell number = 250 ± 40.9 × 10^3^/well, which is 10.4-fold more than H-MOs; Figure 1a); these findings indicated that the presence of HSCs inhibited the propagation of DCs but promoted the generation of CD11b^+^CD11c^−^ cells. Compared with DCs, H-MOs expressed lower levels of CD40, CD86, and IAb (MHC II), a relatively higher level of F4/80, and higher levels of B7-H1 and Gr-1 on the cell surface. The low expression of costimulatory molecules, such as CD40, CD86, and MHC II, indicated that H-MOs were in a more immature stage and less able to induce T-cell activation. In contrast to DCs that produced a high IL-12 level, H-MOs secreted a considerably low IL-12 level but a high IL-10 level (Figure 1b).

To examine the effects of H-MOs on the differentiation and functions of T-cells, a T-cell proliferation assay was performed, and cytokine production was examined. CFSE-labeled BALB/c spleen T-cells were cocultured with H-MOs or DCs at a ratio of 20:1 for 3 days. The proliferation of T-cells and regulatory T-cells was determined using CFSE dilution and a CD4^+^/CD25^+^/Foxp3^+^ marker, respectively, gated in a CD3 population using a flow cytometer. The ability to stimulate T-cell proliferation represents a high capacity to induce host T-cell immunity, whereas the ability to suppress T-cell function represents a high capacity to regulate adaptive immunity. Regulatory T-cells are a subpopulation of T-cells that regulate the immune system and maintain tolerance to self-antigens. H-MOs induced more regulatory T-cells and suppressed the T-cell proliferative response in a dose-dependent manner (Figure 1c). In addition, the production of the cytokine IFN-γ in the culture supernatant or stimulated by allogeneic T-cells indicated that H-MOs attenuated proinflammatory cytokine production (Figure 1d). Taken together, the results demonstrated that the characteristics of H-MOs resemble those of MDSCs with respect to their distinct morphology, low costimulatory molecule levels, decreased proinflammatory cytokine production, and immunosuppressive function on T-cell immunity.

### 2.2. MDSCs Mediated by HSCs Display More Immunoregulatory Enzymes and Regulate T-Cell Activity in the Tumor Environment In Vitro

**Figure 2:**
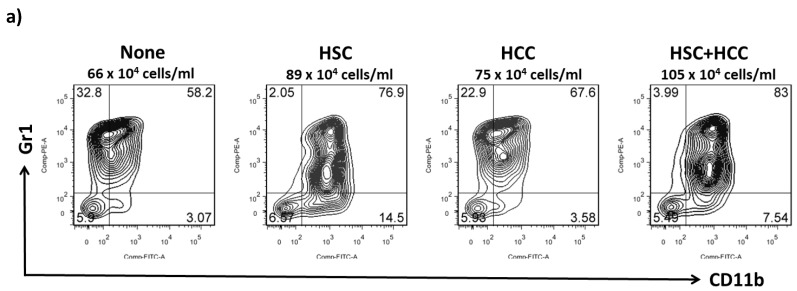

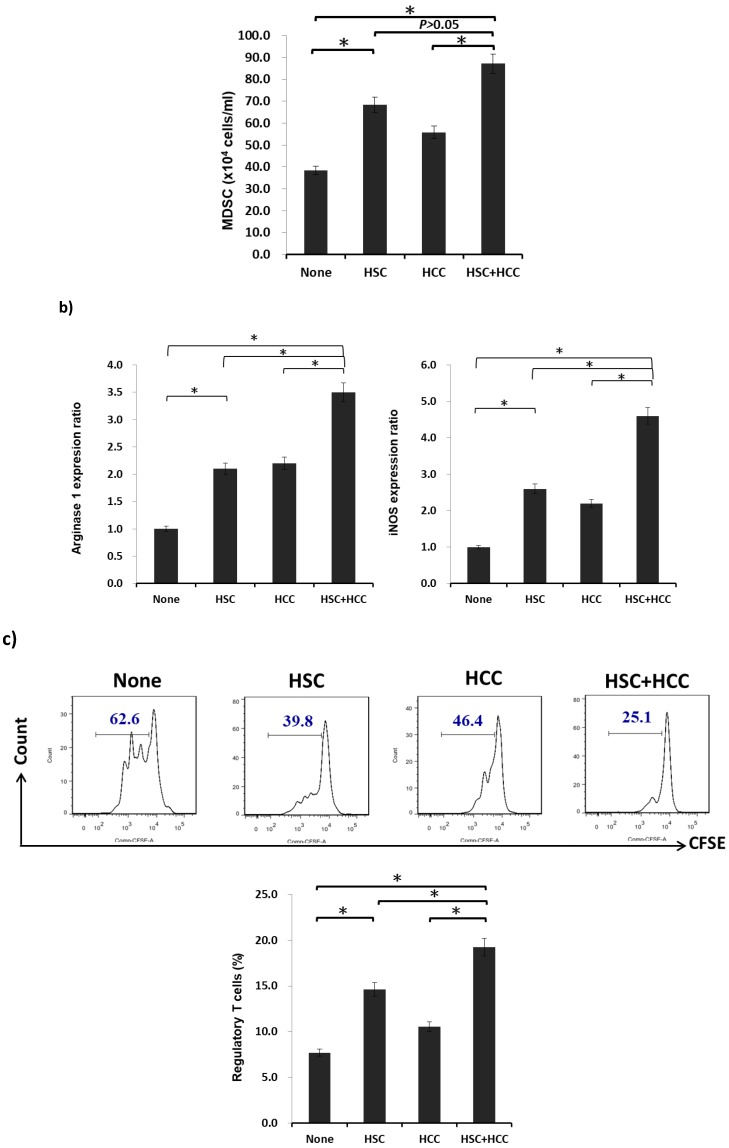

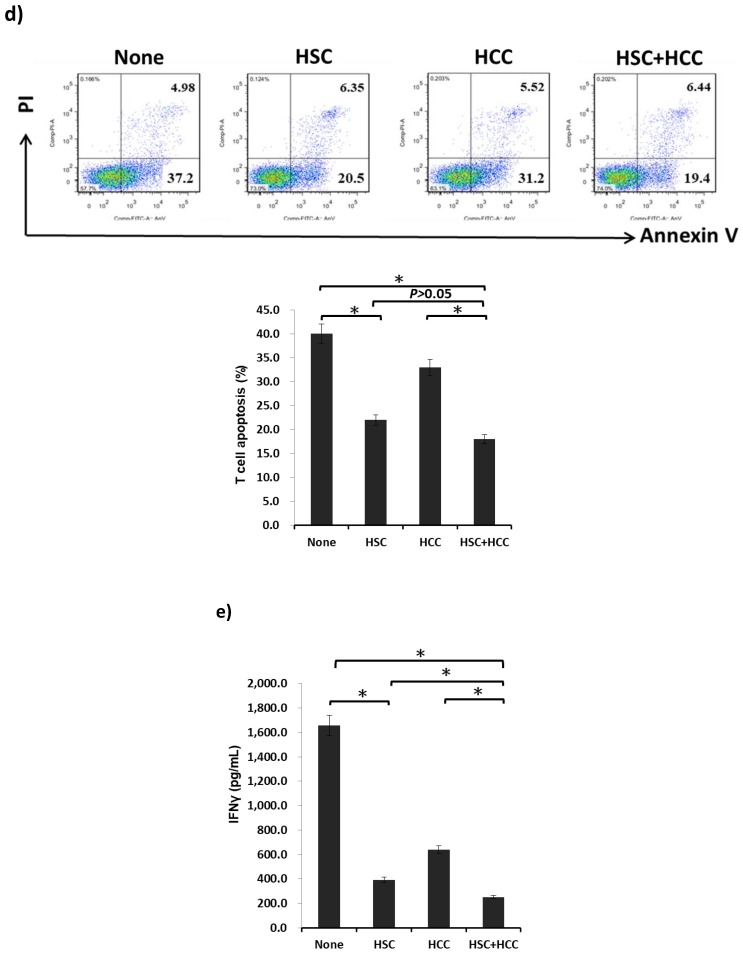
Effect of HSCs on the development of MDSCs in the tumor environment in vitro. HSCs were added into the BM cell culture at a ratio of 1:40 with or without an equal amount of hepatocellular cancer cells (HCCs; Hepa 1-6 cells originate from the mouse hepatoma cell line) in the presence of GM-CSF (8 ng/mL) for five days. (**a**) Cells were stained for CD11b and Gr-1 and analyzed through flow cytometry. Double positivity of CD11b and Gr-1 stands for MDSCs. The absolute numbers of CD11b^+^GR-1^+^ cells/well were calculated and expressed as the mean ± 1 SD (*p* < 0.05). (**b**) Expression of arginase 1 and iNOS mRNA from MDSCs determined using real-time polymerase chain reaction (qPCR) (* *p* < 0.05). Data representative of three separate experiments. (**c**) CFSE-labeled BALB/c spleen T-cells were cultured with MDSCs at a ratio of 20:1 for 3 days. The proliferation of T-cells was determined through CFSE dilution gated in the CD3 population (upper panel). Expression of regulatory T-cells (CD4^+^/CD25^+^/Foxp3^+^) was assayed through intracellular staining with specific mAbs and analyzed through flow cytometry. The bar graph shows the ratio of Treg cells differentiated from four different MDSC groups (lower panel; * *p* < 0.05). (**d**) Activated T-cells were stained for annexin V and propidium iodide (PI) and analyzed through flow cytometry. A positive result for annexin V and a negative result for PI indicate apoptosis (upper panel). The bar graph indicates the ratio of T-cell apoptosis mediated by four MDSC groups (lower panel; * *p* < 0.05). (**e**) Expression of IFN-γ from stimulated allogeneic T-cells was determined in the cultured supernatant by using ELISA (* *p* < 0.05). The data are representative of four separate experiments.

MDSCs are a heterogeneous population of immature myeloid cells that rapidly expand to regulate host immunity during inflammation, infection, and cancer. To examine the effect of HSCs on the differentiation of MDSCs in the tumor environment in vitro, HSCs were added into the BM cell culture at a ratio of 1:40 with or without an equal amount of liver cancer cells (HCCs; Hepa 1-6 cells originated from the mouse hepatoma cell line) in the presence of GM-CSF (8 ng/mL). Five days later, cells were harvested and populations of MDSCs (CD11b^+^Gr-1^+^) and the mRNA expression of iNOS were examined, along with arginase 1 and its effect on T-cell differentiation and functions. The MDSC population was the highest in activated HSCs cocultured with HCCs (87.2 ± 6.9 × 10^4^/well) compared with HSCs (68.4 ± 2.7 × 10^4^/well), HCCs alone (55.8 ± 7.0 × 10^4^/well), or conventional BMs (38.3 ± 2.8 × 10^4^/well; Figure 2a). MDSCs conditioned under the influence of HSCs with HCCs expressed markedly high mRNA levels of iNOS and arginase 1, which play a direct role in the inhibition of immune responses (Figure 2b).

To examine the effects of MDSCs on the regulation of adaptive immunity, particularly T-cells, T-cell differentiation, and cytokine production were evaluated. MDSCs isolated from four groups were added at the beginning into the culture of CFSE-labeled BALB/c splenic T-cells, the proliferation of which was elicited 3 days later. Results demonstrated that MDSCs propagated from HSCs cocultured with HCCs presented the most inhibitory T-cell proliferative response and induced the highest ratio of Treg cells among the four groups (Figure 2c). Apoptosis of T-cells was measured through annexin V and propidium iodide (PI) labeling. Positive annexin V labeling and negative PI labeling represent T-cells that undergo apoptosis. A lower ratio of T-cell apoptosis was induced by MDSCs generated from HSCs cocultured with HCCs compared with HCCs and controls, which showed a relatively lower ratio of T-cell apoptosis than HSCs group (Figure 2d). The lowest level of the proinflammatory cytokine IFN-γ secreted from activated T-cells was observed in the HSC/HCC group (Figure 2e). Collectively, the in vitro results demonstrated that MDSCs mediated by HSCs display more immunoregulatory enzymes, such as iNOS and arginase I, and exhibit potent inhibitory T-cell proliferation activity to regulate host immunity in the tumor environment.

### 2.3. IL-6 Produced by HSCs Participates in the Development of MDSCs In Vitro

**Figure A1:**
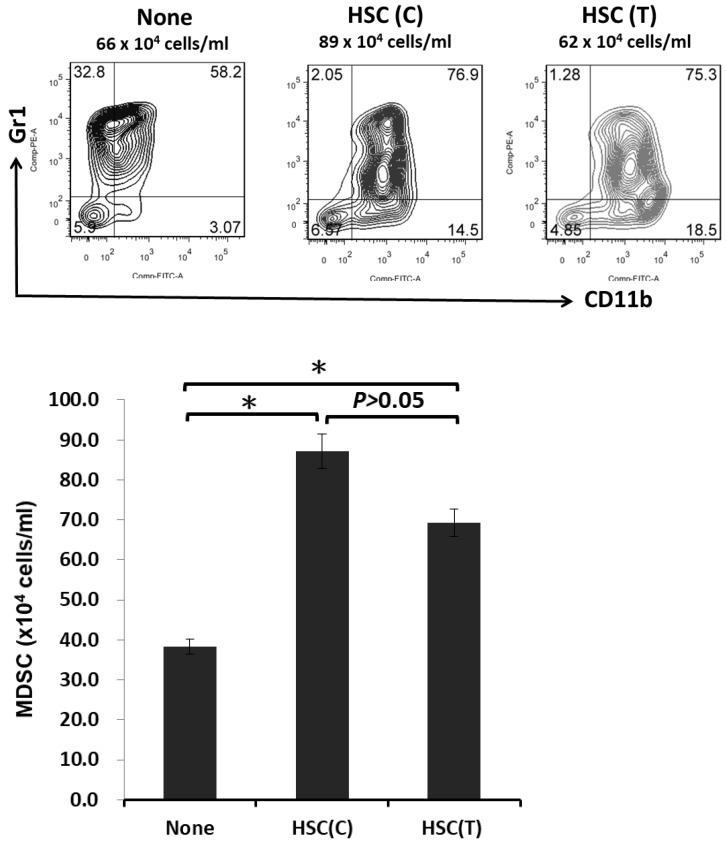
Induction of MDSCs is mediated by soluble factor(s) secreted from HSCs in vitro. BM cells and HSCs were cultured in transwell (T) or conventional plates (C). Expression of CD11b and Gr-1 was analyzed through flow cytometry. Double positivity for CD11b and Gr-1 stands for MDSCs (upper panel). The bar graph shows the absolute numbers of CD11b^+^GR-1^+^ cells/well that were calculated and are expressed as the mean ± 1 SD (* *p* < 0.05; lower panel). Data representative of three separate experiments.

To examine whether the induction of MDSCs is mediated by soluble factors released by HSCs or by cell–cell direct contact, BM cells and HSCs were cultured in transwell plates that blocked cell–cell direct contact but allowed free communication of soluble factors secreted from HSCs. The generation of MDSCs was slightly lower in transwell plates compared with the culture in conventional plates, indicating that soluble factors secreted from HSCs, but not T-cell–cell contact, play a pivotal role in the induction of MDSCs (Appendix A, Figure A1). Activation of HSCs induced the upregulation of IL-6 mRNA transcription [6].

**Figure 3:**
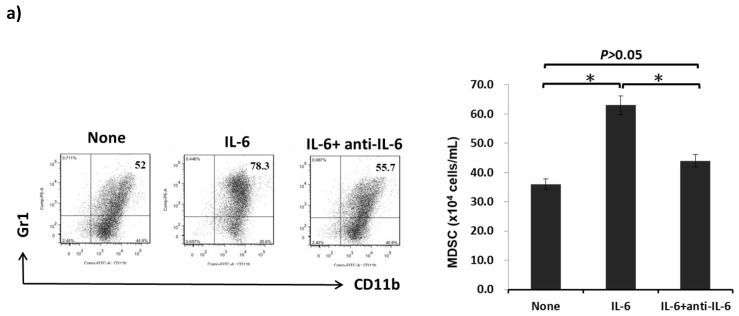

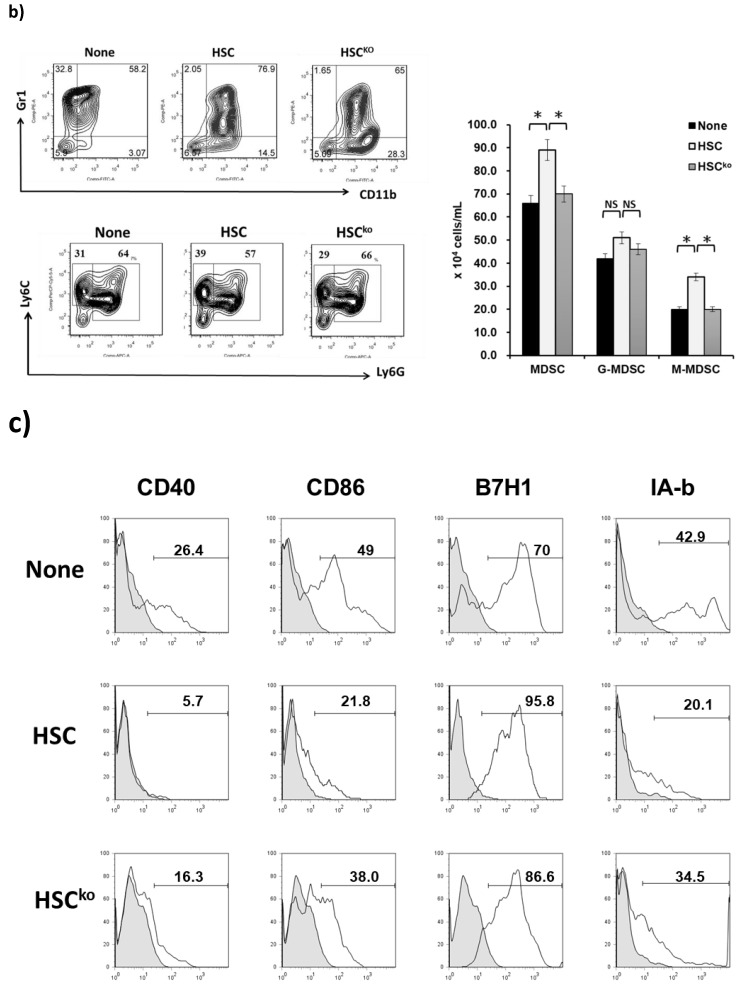

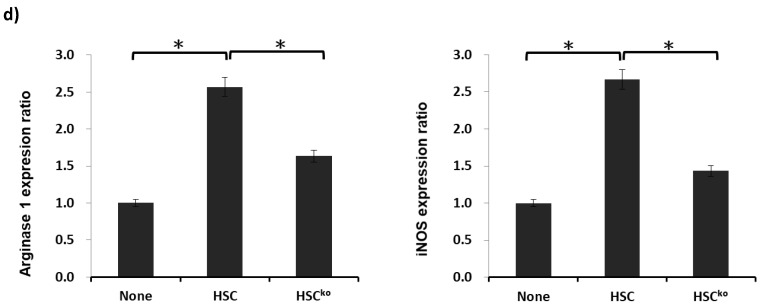
Effect of IL-6 produced by HSCs on the development of MDSCs in vitro. (**a**) B6 BM cells (2 × 10^6^ cells/well) were cultured with recombinant mouse IL-6 (10 ng/mL, BioLegend, San Diego, CA, USA) with or without the anti-IL-6 antibody (10 ng/mL, MP5-20F3, BioLegend, San Diego, CA, USA) in the presence of GM-CSF (8 ng/mL) for 5 days. The cell population expressing the surface molecules CD11b and Gr-1 was analyzed through flow cytometry. Double positivity for CD11b and Gr-1 stands for MDSCs. The bar graph indicates the absolute numbers of CD11b^+^GR-1^+^ cells/well, which are expressed as the mean ± 1 SD (* *p* < 0.05). (**b**) HSCs isolated from wild-type or IL-6-deficient (HSC^ko^) mice were added into the BM cell culture at a ratio of 1:40 in the presence of GM-CSF (8 ng/mL) for 5 days. Cells were stained for CD11b, Gr-1, Ly6C, and Ly6G and analyzed through flow cytometry. Double positivity for CD11b and Gr-1 stands for granulocytic MDSCs (G-MDSCs) with a CD11b^+^Ly6G^+^Ly6C^low^ phenotype and monocytic MDSCs (M-MDSCs) with a CD11b^+^Ly6G^-^Ly6C^high^ phenotype. The bar graph shows the absolute numbers of MDSCs, G-MDSCs, and G-MDSCs per well, which are expressed as the mean ± 1 SD (* *p* < 0.05). (**c**) MDSCs cultured from HSCs or HSC^ko^ were stained for CD40, CD86, IAb (MHC II), and B7-H1 and analyzed through flow cytometry. The flow histograms demonstrate the expression of the indicated surface molecules. (**d**) Expression of arginase 1 and iNOS mRNA from MDSCs cultured from HSCs or HSC^ko^ determined using qPCR (* *p* < 0.05). Data representative of three separate experiments.

To determine the effects of cytokine IL-6 on the generation of MDSCs, B6 BM cells were cultured with IL-6 with or without the anti-IL-6 antibody in the presence of GM-CSF for 5 days. The ratio and numbers of MDSCs were enhanced under the influence of IL-6; however, the effect of IL-6 on the generation on MDSCs reversed after the addition of a neutralized antibody for IL-6 (Figure 3a). These results demonstrated that IL-6 plays a crucial role in the generation of MDSCs.

HSCs isolated from IL-6 knockout mice were utilized to determine the role of IL-6 produced by HSCs in the induction of MDSCs. Wild-type or IL-6 knockout HSCs (HSC^ko^) were added into the BM cell culture for 5 days. The addition of HSC^KO^ significantly reduced MDSC development compared with wild-type HSCs (Figure 3b, left upper plot). MDSCs consist of two major subsets: granulocytic MDSCs (G-MDSCs) with a CD11b^+^Ly6G^+^Ly6C^low^ phenotype and monocytic MDSCs (M-MDSCs) with a CD11b^+^Ly6G^−^Ly6C^high^ phenotype. HSCs isolated from wild-type mice mainly increased the ratio and number of M-MDSCs rather than those of G-MDSCs compared with IL-6-deficient HSCs (Figure 3b, left lower and right plot). Wild-type HSC-induced MDSCs expressed lower levels of CD40, CD86, and IAb (MHC II), and higher levels of B7-H1 compared with HSC^ko^-induced MDSCs (Figure 3c). The expression of iNOS and arginase 1 in MDSCs mediated by wild-type HSCs was significantly decreased when conditioned with HSCs lacking IL-6 (Figure 3d). Taken together, the in vitro results demonstrated that IL-6 produced by HSCs participates in the development of MDSCs (mainly M-MDSCs), and expresses more immunoregulatory activity.

### 2.4. IL-6 Secreted from HSCs Expands the Population of MDSCs to Promote HCC Growth In Vivo

**Figure 4:**
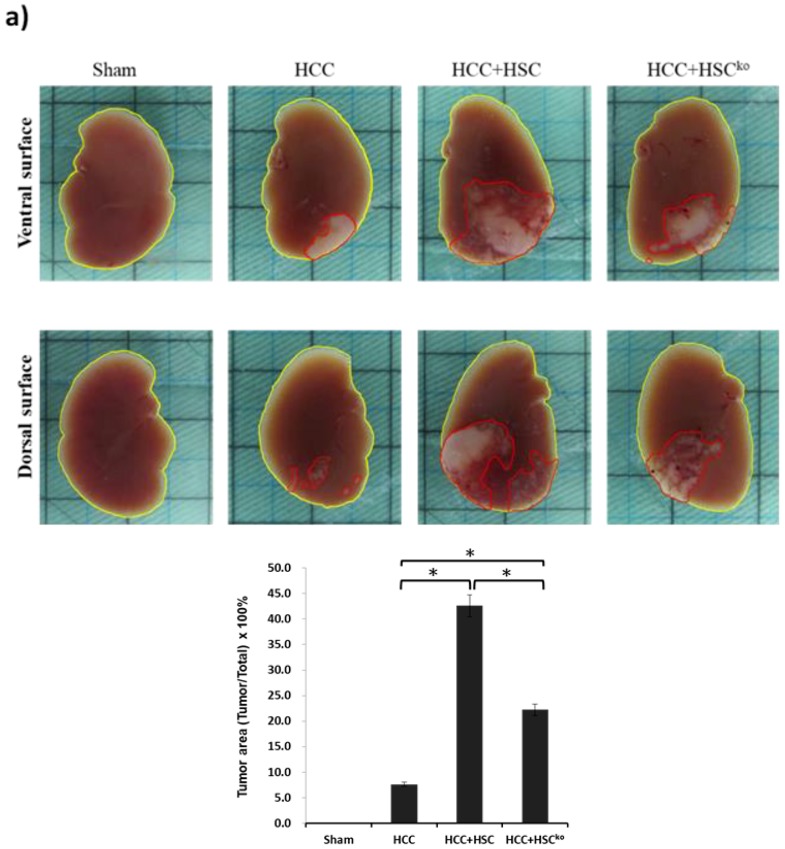

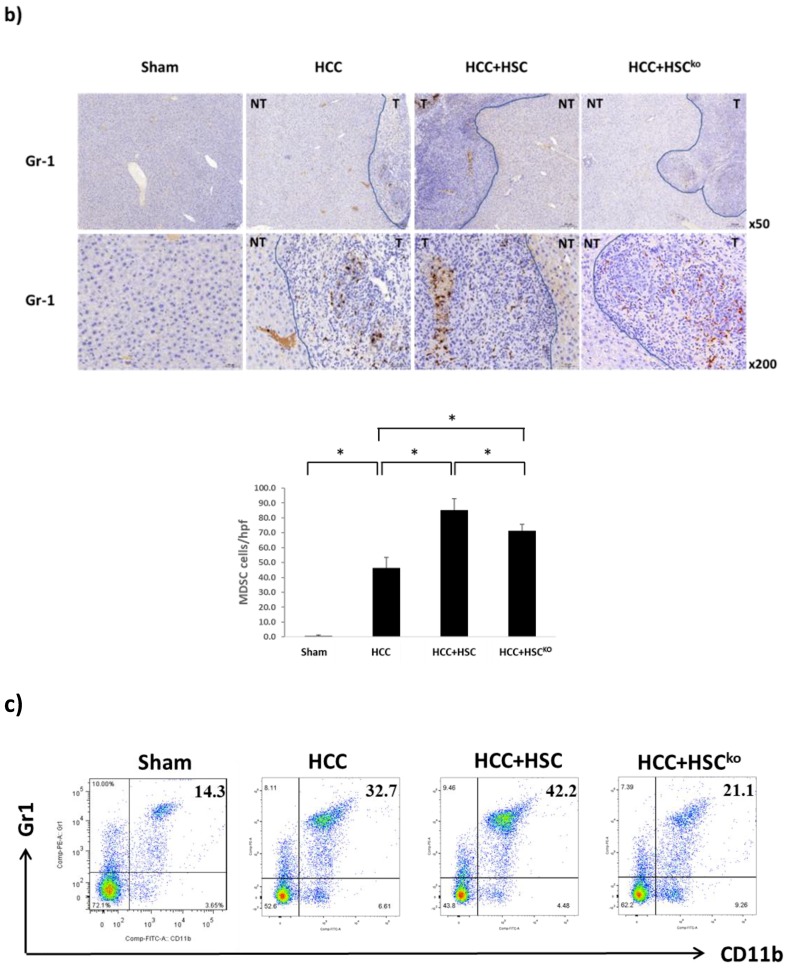

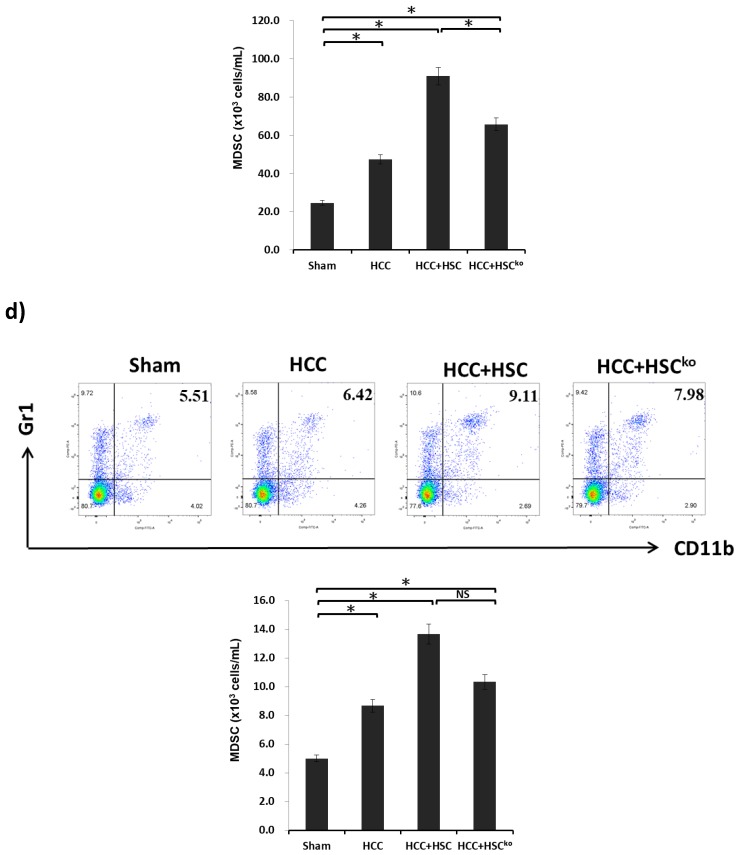
IL-6 secreted from HSCs expand the population of MDSCs to promote HCC growth in vivo. (**a**) An orthotopic liver tumor inoculation model. Liver cancer cells (HCCs; Hepa 1-6 cell lines, 1 × 10^6^ cells per mouse) were implanted into the left liver with or without HSCs (3.3 × 10^5^ cells per mouse) after being harvested from wild-type or IL-6 knockout mice. The sham group, as the control, received implantation of the same volume of PBS (*n* = 5). The other three groups, namely HCC, HCC+HSC, and HCC+HSC^KO^, were conducted with 5 mice in each study. The liver was harvested after 14-day implantation. The tumor area, including the ventral and dorsal surfaces of the liver, was calculated using ImageJ software. The bar graph shows the HCC tumor area among the four groups, which was expressed as the mean ± 1 SD (* *p* < 0.05). The data are representative of three separate experiments. (**b**) Liver cryostat sections were histochemically stained with anti-Gr-1 mAb (1:50, RB6-8C5, BioLegend, San Diego, CA, USA), and were examined by microscopy. The transition zone between the tumor (T) and non-tumor (NT) area were presented with low-power (x50) and high-power magnification (x200). The bar graph illustrates the number of MDSCs counted in a total of 10 high-power fields randomly selected in each section (lower panel; * *p* < 0.05). (**c**) Blood was drawn for staining for CD11b and Gr-1 in four groups after 14-day implantation. Cells were stained for CD11b and Gr-1 and analyzed through flow cytometry. Numbers in the histogram represent the percentage of MDSCs. The absolute numbers of CD11b^+^GR-1^+^ cells/well were calculated and expressed as the mean ± 1 SD (* *p* < 0.05). (**d**) The spleen was harvested and digested for single-cell suspension after 14-day implantation. Cells were stained for CD11b and Gr-1 and analyzed through flow cytometry. Numbers in the histogram represent the percentage of MDSCs. The absolute numbers of CD11b^+^GR-1^+^ cells/well were calculated and expressed as the mean ± 1 SD (* *p* < 0.05, NS: not significant). Data representative of three separate experiments.

To examine whether IL-6 participates in the immunoregulatory capacity of MDSCs mediated by HSCs, liver cancer cells (HCCs, Hepa 1-6 cell line, 1 × 10^6^ cells per mouse) were implanted into the left liver with or without HSCs (3.3 × 10^5^ cells per mouse) harvested from wild-type or IL-6 knockout mice by using the orthotopic liver tumor inoculation model. The tumor area was calculated using the ImageJ method after 14-day implantation. HCCs cotransplanted with HSCs significantly increased the tumor area from 7.6% to 42.6% compared with HCCs alone (42.6% ± 5.1% vs. 7.6% ± 1.4%, *p* < 0.05). By contrast, HCCs cotransplanted with IL-6-deficient HSCs reduced the tumor area from 42.6% to 22.2% compared with HCCs cotransplanted with wild-type HSCs (22.2% ± 3.9% vs. 42.6% ± 5.1%, *p* < 0.05; Figure 4a). These results revealed that IL-6 secreted from HSCs can enhance HCC progression.

MDSCs possess immunoregulatory properties and participate in tumorigenesis, immune tolerance, and infection. As illustrated in Figure 4b, the number of MDSCs within the liver tumor area was higher in three HCC-bearing mice, namely those bearing HCCs alone, HCCs cotransplanted with HSCs, and HCCs cotransplanted with IL-6-deficient HSCs, compared with non-tumor-bearing mice (sham group). HCCs cotransplanted with wild-type HSCs induced the highest level of MDSCs within the liver tumor area among the three HCC-bearing mice. In addition, the tumor area of HCC was strongly associated with the population of MDSCs. The number of MDSCs in blood was higher in the three HCC-bearing mice groups than in the sham group. The group of HCCs cotransplanted with HSCs generated a higher ratio and number of MDSCs than did the group of HCCs cotransplanted with IL-6-deficient HSCs (Figure 4c, *p* < 0.05). The population of MDSCs in the spleen still increased in HCC-bearing mice, but the number of MDSCs did not exhibit a significant difference between HCCs cotransplanted with HSCs and HCCs cotransplanted with IL-6-deficient HSCs (Figure 4d). Collectively, these results indicate that IL-6 secreted from HSCs can expand the population of MDSCs within the tumor and blood to promote HCC growth.

## 3. Discussion

The liver is an immune-privileged organ because of its anatomical location and function, and is continuously exposed to various antigens, including dietary and commensal antigens [17]. Thus, the liver contains cells that have potent immunoregulatory activities to control inappropriate immune responses to those foreign antigens. HSCs are thought to play a crucial role in regulating immune responses. Cotransplantation with HSCs effectively prolonged the survival of islet allografts through the induction of effector T-cell apoptosis and the generation of MDSCs and Treg cells [6,8,18,19]. In our previous study, we reported that HSCs promoted the generation of MDSCs to regulate immune tolerance mediated by complement 3 (C3), which is a soluble factor secreted from HSCs [9], but C3-deficient HSCs did not totally lose their capacity to generate the population of MDSCs. This finding suggested that in addition to HSCs, other factors may be involved in the propagation of MDSCs.

Yu et al. demonstrated that HSCs activated by a 7-day culture induced the upregulation of IL-6 and TGF-β and the mRNA transcription of the migration inhibitory factor. Further stimulation by IFN-γ or syngeneic or allogeneic T-cells did not further upregulate cytokine expression in activated HSCs [6]. Wang et al. demonstrated that the plasma IL-6 level of HCC patients was associated with tumor progression and postoperative tumor relapse. In addition, IL-6 could enhance the stemness and promote the metastasis of HCCs in vitro and in vivo [20], suggesting the importance of IL-6 in HCC progression. The current study results demonstrated that IL-6 produced by HSCs is critical in the generation of MDSCs in vivo and in vitro. MDSCs conditioned by wild-type B6 HSCs expressed low CD40, CD86, and MHC II levels and higher B7-H1 levels than did those mediated by IL-6-deficient HSCs. HSCs increased the population of M-MDSCs rather than that of G-MDSCs through IL-6. In addition, the expression of inhibitory enzymes, namely iNOS and arginase I, in MDSCs was increased when conditioned by wild-type HSCs compared with when conditioned with HSCs lacking IL-6. Xu et al. reported that activated HSCs promote liver cancer by means of MDSCs through cyclooxygenase-2 rather than IL-6. The authors reported that IL-6-neutralizing antibody used to deactivate IL-6 in the HSC culture medium did not reduce the population of MDSCs [21]. In the current study, we utilized the IL-6-deficient mouse model to elucidate that IL-6 secreted from HSCs definitively mediated the development of MDSCs.

MDSCs are a heterogeneous population of immature myeloid cells that rapidly expand to regulate host immunity during inflammation, infection, and cancer [22]. The populations of MDSCs expand in the tumor site, lymphoid tissues (including the spleen), and peripheral blood in tumor-bearing mice and in patients with cancer. Several studies have demonstrated that the MDSC population is influenced by tumor cells that promote the expansion of MDSCs through the stimulation of myelopoiesis and the inhibition of the differentiation of mature myeloid cells and tumor stroma, or activated T-cells that are directly involved in activating MDSCs [23,24,25,26]. Factors that induce MDSC expansion include cyclooxygenase-2, prostaglandins [27], stem-cell factor [28], macrophage colony-stimulating factor, IL-6 [25], GM-CSF [29], and vascular endothelial growth factor [30]. In the current study, the phenomenon whereby the population of MDSCs expands in the tumor, spleen, and peripheral blood in tumor-bearing mice was verified using three HCC-bearing mice models, namely those bearing HCCs alone, HCCs cotransplanted with HSCs, and HCCs cotransplanted with IL-6-deficient HSCs. The tumor area of HCCs was strongly and positively associated with the number of MDSCs present within the tumor. The MDSC population is influenced by liver cancer cells (HCCs) and augmented with liver stromal cells (HSCs), as well as IL-6 secreted from HSCs. The limitation of the current in vivo study is that it cannot provide direct evidence to verify that HCC promotion is enhanced by the population of MDSCs induced by HSCs through IL-6 secretion. Wang et al. demonstrated that the level of IL-6 is strongly associated with HCC progression [20]. In addition, HSCs produced more IL-6 mRNA when activated [6]. The pivotal role of IL-6, secreted from HSCs or the tumor environment, may entail direct enhancement of HCC growth rather than induction of MDSCs.

Immune suppression is a principal feature of MDSCs. Although MDSCs are involved in the suppression of various cells of the immune system, the main targets of MDSCs are T-cells. MDSCs inhibit T-cell immunity through various mechanisms, including the expression of iNOS and arginase I [31,32,33], and the production of nitric oxide, reactive oxygen species, and peroxynitrite [34]. In the current study, MDSCs mediated by HSCs cocultured with HCCs expressed markedly higher levels of iNOS and arginase I than those mediated by HSCs alone. Furthermore, MDSCs propagated from HSCs cocultured with HSCs displayed a more inhibitory T-cell proliferative response, a higher ratio of Treg cells, a lower level of T-cell apoptosis, and a lower production of IFN-γ from stimulated T-cells compared with those propagated from HSCs alone. These data demonstrated that MDSCs conditioned by HSCs produce more immunoregulatory enzymes to inhibit T-cell immunity, especially under the influence of the HCC environment.

Taken together, the current study demonstrated that the generation of MDSCs is influenced by liver cancer cells and hepatic stellate cells. MDSCs are induced mainly by HSCs through IL-6 signaling and produce inhibitory enzymes to reduce T-cell immunity and then promote HCC progression.

## 4. Materials and Methods

### 4.1. Animals and Cell Line

Male C57BL/6 (B6) and BALB/c mice were purchased from the National Laboratory Animal Center (Taiwan). IL-6 knockout mice on a C57BL/6J background (B6.129S2-Il6tm1Kopf/J) were purchased from Jackson Laboratory (Bar Harbor, ME, USA). All animal experiments were approved by the Institutional Animal Care and Use Committee of Chang Gung Memorial Hospital (IACUC permit number: 2014123001, date of approval on 1 March 2015). The Hepa 1-6 cell line, which is a mouse hepatoma cell line derived from ATCC CRL-1830 and obtained from the Bioresource Collection and Research Center (Hsinchu city, Taiwan), was used in this study.

### 4.2. Preparation of HSCs

HSCs were isolated from mouse livers, as described previously [6,35]. Briefly, the livers were perfused with phosphate-buffered saline (PBS) and type IV collagenase and soaked in collagenase for further digestion. HSCs were isolated and enriched through Percoll gradient centrifugation and cultured (10^5^ cells/mL) in cell culture flasks (25-cm^2^ surface area) with RPMI-1640 supplemented with 20% vol/vol heat-inactivated fetal calf serum in 5% CO2/95% air at 37 °C for 7–10 days. Cell viability was greater than 90%, as determined using the trypan blue exclusion test. The purity of HSCs was determined to be >95%, on the basis of desmin immunostaining results and the typical light microscopic appearance of lipid droplets.

### 4.3. Culture of DCs, MDSCs, and HSC-Conditioned MDSCs

DCs were prepared as described previously [36,37]: 2 × 10^6^/well bone marrow (BM) cells from the tibias and femurs of B6 mice were cultured in RPMI-1640 medium containing 10% fetal calf serum in the presence of mouse recombinant granulocyte/macrophage colony-stimulating factor (GM-CSF; 8 ng/mL, R&D Systems, Minneapolis, MN, USA) for 5 days. Cells that were double positive for CD11b and CD11c were considered DCs, and those that were double positive for CD11b and Gr-1 were considered MDSCs. To examine the effect of IL-6 produced by HSCs on the production of MDSCs, WT, or IL-6-deficient HSCs were added at the beginning of the culture at an HSC/BM cell ratio of 1:40. To create the tumor environment, HCCs were added at the beginning of the culture at an HCC/HSC cell ratio of 1:1.

### 4.4. Orthotopic Hepatic Tumor Inoculation Model

The tumor model was created through the direct intrahepatic injection of mouse hepatoma cells (Hepa 1-6). Mice were anesthetized, and a midline incision was made to expose the liver. Hepa 1-6 cells alone (1 × 10^6^ cells originating from C57BL/6 mice) or cotransplanted with 3.3 × 10^5^ HSCs from wild-type or IL-6 knockout mice were resuspended in 30–50 µL of PBS. Cells were then slowly injected under the hepatic capsule into the upper left lobe of the liver using a 29-G needle. A pale whitish color could be observed at the point of injection under the hepatic capsule. Gentle compression was applied for 15 s with a cotton applicator to prevent bleeding and reflux of cells. The abdomen was closed with a 6–0 silk suture. Mice were observed for 2–3 h and then returned to housing facilities.

### 4.5. Tumor Area Calculation

The tumor area of the liver was analyzed using ImageJ^®^ software, a Java-based image processing software developed by the National Institute of Health (Bethesda, MD, USA). A score for regions of interest (ROIs) in the analyzed field was calculated using ImageJ software. The tumor area index was calculated as follows: Tumor Area Index = (ROIs of the Tumor/Total ROIs) × 100% [38].

### 4.6. Flow Cytometry Analysis

Monoclonal antibodies (mAbs) against CD4 (1:200, GK1.5), CD11b (1:200, M1/70), CD11C (1:200, HL3), CD25 (1:200, PC61), CD80 (1:200, 16-10A1), CD86 (1:200, GL1), and Gr-1 (1:200, RB6-8C5); and I-Ab (MHC class II) (1:200, AF6-120.1), IFN-γ (1:200, XMG1.2), propidium iodide (PI) (1:200), annexin V (1:200), and Ly6-G (1:200, 1A8), B7-H1 (1:200, MIH5) were purchased from BD Biosciences (San Jose, CA, USA). The mABs against CD3 (1:200, 17A2), CD40 (1:200, 3/23), F4/80 (1:200, BM8), and Ly6-C (1:200, HK1.4) were purchased from BioLegend (San Diego, CA, USA). Foxp3 (1:200, FJK-16s) was purchased from Invitrogen (San Diego, CA, USA). Intracellular staining protocols for regulatory T-cells were followed for Foxp3 staining. For carboxyfluorescein succinimidyl ester (CFSE) labeling, splenic T-cells (10^7^/mL) from BALB/c mice were incubated with 0.5 µM CFSE (Invitrogen, San Diego, CA, USA) for 10 min at room temperature. A flow analysis was performed using the BD FACSCanto II flow cytometer (BD Bioscience, Franklin Lakes, NJ, USA).

### 4.7. Quantitative Reverse Transcription Polymerase Chain Reaction

Total RNA was extracted using an RNeasy mini kit (Qiagen, Valencia, CA, USA). RNA samples were first converted into cDNA by using a RevertAid First Strand cDNA Synthesis Kit (Thermo Fisher, Waltham, MA, USA). Primers used in quantitative polymerase chain reaction (PCR) for arginase-1 and inducible nitric oxide synthase (iNOS) were as follows: forward, CACGG CAGTG GCTTT AACCT; reverse, TGGCG CATTC ACAGT CACTT; forward, TGGCC ACCTT GTTCAG CTACG; and reverse, GCCAA GGCCA AACAC AGCAT AC, respectively. mRNAs were measured using a CFX96 Touch Real-Time PCR system (Bio-Rad Laboratories, Inc, Hercules, CA, USA) in duplicate and were normalized to 18S mRNA.

### 4.8. Immunohistochemistry Staining

Gr-1 expression of the liver tumor area in cryostat sections was identified through fluorescence staining using a specific anti-Gr-1 antibody (1:50, RB6-8C5, BioLegend, San Diego, CA, USA), following permeabilization with 0.05% saponin buffer by using a Vectastain Elite ABC kit (Vector Lab, Inc, Burlingame, CA, USA) as immunoperoxidase. Slides were developed using 3-amino-9-ethylcarbazole (AEC) chromogen/substrate and counterstained with hematoxylin. Isotype- and species-matched irrelevant antibodies served as controls. For quantification, cells were counted under a microscope, and a total of 10 high-power fields were randomly selected.

### 4.9. Statistical Analyses

Statistical analyses were performed using Student’s *t* test for independent samples, with significance determined at *p* < 0.05. All data, means, and standard deviations (SDs) were calculated and graphed in Microsoft Excel (Microsoft, Redmond, WA, USA).

## 5. Conclusions

MDSCs are induced mainly by HSCs through IL-6 signaling and produce inhibitory enzymes to reduce T-cell immunity and then promote HCC progression. Therapies targeting the involved molecules can serve as an alternative immunotherapy protocol for HCC.

## Abbreviations

B7H1	B7 homolog 1
BM	Bone marrow
C3	Complement factor 3
CD	Cluster of differentiation
CFSE	Carboxyfluorescein succinimidyl ester
DC	Dendritic cell
ELISA	Enzyme-linked immunosorbent assay
FOXp3	Forkhead box p3
GM-CSF	Granulocyte-macrophage colony-stimulating factor
G-MDSC	Granulocytic MDSC
HCC	Hepatocellular carcinoma
H-MO	HSC-conditioned monocyte
HSC	Hepatic stellate cell
HSC^ko^	IL-6 knockout HSC
IFNγ	Interferon gamma
IL	Interleukin
IMC	Immature myeloid cells
iNOS	Inducible NO synthase
mAb	Monoclonal Ab
MDSC	Myeloid-derived suppressor cell
M-MDSC	Monocytic MDSC
PI	Propidium iodide
ROI	Regions of interest
Treg	Regulatory T-cell
WT	Wild type
